# Molecular basis of carrageenan-induced cytokines production in macrophages

**DOI:** 10.1186/s12964-020-00621-x

**Published:** 2020-09-07

**Authors:** Alexandre H. Lopes, Rangel L. Silva, Miriam D. Fonseca, Francisco I. Gomes, Alexandre G. Maganin, Lucas S. Ribeiro, Lucas Maciel Mauriz Marques, Fernando Q. Cunha, Jose C. Alves-Filho, Dario S. Zamboni, Norberto P. Lopes, Bernardo S. Franklin, Aurélie Gombault, Fernando Silva Ramalho, Valerie F. J. Quesniaux, Isabelle Couillin, Bernhard Ryffel, Thiago M. Cunha

**Affiliations:** 1 Department of Pharmacology, Ribeirão Preto Medical School, University of São Paulo, Center for Research in Inflammatory Diseases (CRID)Av. Bandeirantes 3900, 14049-900, Ribeirão Preto, SP Brazil; 2 Institute of Innate Immunity, University Hospitals, University of Bonn, 53127 Bonn, Germany; 3 Department of Physics and Chemistry, University of São Paulo, Ribeirão Preto, SP Brazil; 4 Department of Cellular and Molecular Biology, Ribeirão Preto Medical School, University of São Paulo, Ribeirão Preto, SP Brazil; 5 University of Orleans and CNRS, UMR7355 Experimental and Molecular Immunology, Orleans, France; 6 Department of Pathology, School of Medicine of Ribeirão Preto, University of São Paulo, Ribeirão Preto, SP Brazil

**Keywords:** Carrageenan, Macrophages, IL-1β, NLRP3 Inflammasome, Pannexin-1 channel

## Abstract

**Background:**

Low molecular weight carrageenan (Cg) is a seaweed-derived sulfated polysaccharide widely used as inflammatory stimulus in preclinical studies. However, the molecular mechanisms of Cg-induced inflammation are not fully elucidated. The present study aimed to investigate the molecular basis involved in Cg-induced macrophages activation and cytokines production.

**Methods:**

Primary culture of mouse peritoneal macrophages were stimulated with *Kappa* Cg. The supernatant and cell lysate were used for ELISA, western blotting, immunofluorescence. Cg-induced mouse colitis was also developed*.*

**Results:**

Here we show that Cg activates peritoneal macrophages to produce pro-inflammatory cytokines such as TNF and IL-1β. While Cg-induced TNF production/secretion depends on TLR4/MyD88 signaling, the production of pro-IL-1β relies on TLR4/TRIF/SYK/reactive oxygen species (ROS) signaling pathway. The maturation of pro-IL1β into IL-1β is dependent on canonical NLRP3 inflammasome activation via Pannexin-1/P2X7/K^+^ efflux signaling. In vivo, Cg-induced colitis was reduced in mice in the absence of NLRP3 inflammasome components.

**Conclusions:**

In conclusion, we unravel a critical role of the NLRP3 inflammasome in Cg-induced pro-inflammatory cytokines production and colitis, which is an important discovery on the pro-inflammatory properties of this sulfated polysaccharide for pre-clinical studies.

**Video abstract**

**Graphical Abstract:**

Carrageenan (Cg) is one the most used flogistic stimulus in preclinical studies. Nevertheless, the molecular basis of Cg-induced inflammation is not totally elucidated. Herein, Lopes et al. unraveled the molecular basis for Cg-induced macrophages production of biological active IL-1β. The Cg-stimulated macrophages produces pro-IL-1β depends on TLR4/TRIF/Syk/ROS, whereas its processing into mature IL-1β is dependent on the canonical NLRP3 inflammasome. 
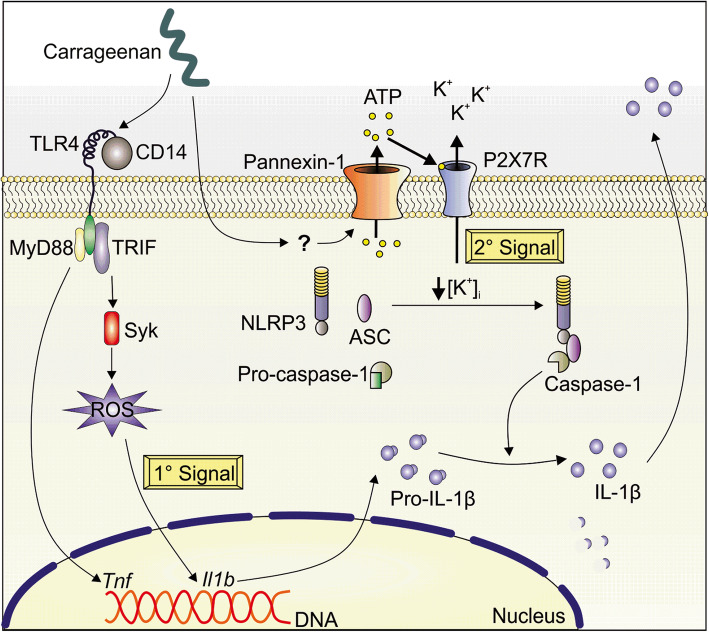

## Background

Carrageenan (Cg) is a seaweed-derived sulfated polysaccharide [1, 2]. There are three major forms of carrageenan: lambda- (*λ*), kappa- (*κ*) and iota- (*ι*) [3, 4]. There is evidence that chronic ingestion of some subtypes of Cg, especially low-molecular weight, is associated with inflammation, intestinal cancer and ulcerations [5–7]. Furthermore, these flogistic Cg subtypes are extensively used to induce inflammation in several experimental animal models especially to study novel anti-inflammatory and analgesic drugs [8–11]. For instance, Cg injected into the rodents hindpaw causes a classical innate immune response characterized by paw oedema, neutrophil migration and pain [12, 13].

Our group has identified that Cg-induced inflammation and inflammatory symptoms are dependent on activation of macrophages through the production of pro-inflammatory cytokines, especially TNF and IL-1β [14–16]. In vitro, Cg induces peritoneal macrophages activation and the production of these pro-inflammatory cytokines [17, 18]. In regards to the possible molecular mechanisms, there is evidence that seaweed-derived polysaccharides can regulate the innate immune response directly through the binding of pattern recognition receptors (PRRs), such as mannose and Toll-like receptors (TLR) in phagocytic cells, including macrophages [19–22]. Precisely, Cg activates TLR4 in vitro [18, 23, 24] and induces processing of pro-IL-1β into mature IL-1β dependently on caspase-1 (Casp1) activation in vivo [25].

However, the exact molecular mechanisms of Cg-induced macrophages activation and pro-inflammatory cytokines production remains to be demonstrated. Herein, we set out to clarify the molecular mechanisms involved in macrophages activation by Cg, focusing on the intracellular pathways for IL-1β production/release. We demonstrate that peritoneal macrophages from naïve mice produce both forms of pro-inflammatory IL-1β when stimulated with Cg alone. Furthermore, we provided evidence that Cg-stimulated macrophages produce pro-IL-1β depends on TLR4/TRIF/Syk/ROS, whereas it’s processing into mature IL-1β requires activation of the canonical NLRP3 inflammasome. Finally, we show that NLRP3 inflammasome is implicated in Cg-induced colitis in mice. These data unravel the previous unknown molecular basis for IL-1β production by Cg-stimulated macrophages and in vivo inflammation.

## Methods

### Animals

The experiments were performed using six to eight-week-old-male weight (25 g) wild type (WT) C57BL/6 mice (Jackson Laboratory, stock number 000664) and deficient (^−/−^) mice in the following genes: *Nlrp3*^−/−^, *Casp1/11*^−/−^, *Casp1*^*−/−*^*Casp11Tg*, *Nlrc4*^−/−^, *Pycard*^−/−^ (ASC) derived from C57BL/6 mouse strains. The ASC-mCitrine reporter mice were generated following a strategy previously described [26]. *Tlr2*^−/−^, *Tlr3*^−/−−^, *Tlr4*^−/−^, *Tlr5*^−/−^, *Cd14*^−/−^, *Trif*^−/−^, *Myd88*^−/−^. *Panx1*^−/−^ mice were kindly provided by Dr. Valery Shestopalov (University of Miami). All strains were maintained at the animal facility of the Ribeirão Preto Medical School, University of São Paulo (São Paulo, Brazil). *P2rx7* ^−/−^ mice were obtained from the Immunology and Experimental Neurogenetics at the National Center for Scientific Research (CNRS) University of Orléans - France by Dr. Bernhard Ryffel and Dra. Isabelle Couillin. Breeding pairs of mice with targeted disruptions of all genes were maintained/backcrossed with C57BL/6. Animal care and handling procedures were performed in accordance with the current guidelines in accordance with Ethics Committee on Animal Experimentation of the Ribeirão Preto Medical School, University of São Paulo.

### Reagents

The following reagents were used in this study: Lipopolysaccharide (ultrapure LPS), N-Acetyl-L-cysteine (NAC – A7250, Sigma Aldrich), Carbenoxolone (CBX – C4790, Sigma Aldrich), Nigericin (Nig – N7143, Sigma Aldrich). The Kappa-Carrageenan (Cg) was obtained from BDH Biochemicals (Poole, England Cat. n° 38,100). The concentration used for the in vitro experiments range from 30 μg/ml up to 300 μg/ml. KCl (Cat. n°. 7447–40-7, Labsynth), Caspase-1 inhibitor (Calbiochem, Cat. n°. 400,010), Syk inhibitor (Millipore, Cat. n°. 574,711).

### Isolation and culture of peritoneal macrophages

Macrophages were extracted by washing the peritoneal cavity of health animals. The total contents of the peritoneal lavage were centrifuged at 450 g for 8 min, then the cell pellet was resuspended in complete RPMI medium containing 10% fetal bovine serum, 2.38 g/L Hepes (Sigma), 100,000 U/L Penicillin/Gentamycin (Sigma), 1 g/L Streptomycin (Sigma), 2.2 g/L Sodium Bicarbonate PA (Merck). Then cell concentration was determined and subsequently distributed into stereo plates and incubated at 37 °C overnight. Non-adherent and dead cells were removed by gentle washed with RPMI medium. The remaining cultured cells were at least 97% macrophages and trypan blue exclusion test showed viability higher than 95%. The cells were cultured in 9 6-well plate 2 × 10^5^ cells/well for further ELISA assays, or in 6-well plate 2 × 10^6^ cells/well for western blotting. After the treatments the supernatant and the cell lysate were collected and prepared for molecular analysis.

### Enzyme-linked immunosorbent assays (ELISA)

Colon tissue or supernatant from cultured peritoneal macrophages were used to determine the levels of IL-1β (R&D systems, cat. DY401–05) and TNF (R&D systems, cat. DY4 10-05) by ELISA according to the manufacturer instructions. The results were expressed in picograms of the respective cytokine per milliliter.

### MTT assay

Cell viability was determined by MTT assay adapted from Mosmann [27]. After the cell treatments, MTT (Thiazolyl Blue Tetrazolium Bromide – M2128, Sigma-Aldrich) at 2,5 mg/ml was added in the 96-well plate containing the attached macrophages. After overnight incubation at 37 °C, the stain was diluted with 50 μL DMSO/acid acetic and was incubated 2 h at 37 °C. The absorbance of each well was then measured using a microplate reader at 592 nm, and the viability of cells was presented as absorbance value. Each treatment was replicated at least four times.

### Assay for endotoxin detection

ToxinSensor™ Single Test Kit (Cat. N°. L00447–40, GenScript) was used to detect contaminants, such as endotoxins in the carrageenan. Vehicle or samples with different concentrations of the carrageenan were incubated for 1 h with the kit reagent. The presence of endotoxin causes gelation and in the absence of endotoxin no gelation occurs.

### Quantification of reactive oxygen species (ROS)

Dichlorofluorescein diacetate (CM-H2DCFDA, Cat. C6827, Invitrogen) was used as a fluorescent marker to detect the production of reactive oxygen species (ROS). Briefly, 30 min before carrageenan stimulation (T_0_), the isolated cells were incubated with the 5 mM H2DCFDA probe, then after 30 min incubation at 37 °C, the cells were washed twice in PBS. During all procedures, cells were kept protected from light to prevent loss of fluorescence. The results were quantified by using a fluorimeter apparatus (FlexStation 3, Molecular Devices).

### *Il1b* gene expression: mRNA extraction and quantitative Real-time Polymerase Chain Reaction (qRT-PCR)

The *Il1b* mRNA was determined by qRT-PCR. Samples from macrophages were lysed and the total cellular RNA was extracted as instructed by Qiagen’s RNeasy Mini Kit (Invitrogen Life Technologies Corporation) and RNA concentration was determined using Genequant apparatus (Amershan Biosciences Corp., Piscataway). mRNA was reversely transcribed into cDNA using High Capacity cDNA Reverse Transcription kit (Life Technologies). qRT-PCR was performed in Step One Plus Real-Time PCR System using the SYBR-green® fluorescence system (Applied Biosystems) for the quantification of amplifications. The results were analyzed using the comparative method of 2 ΔΔ cycle threshold (CT). The following primer sequences were used: *Gapdh* forward 5′-GGGTGTGAACCACGAGAAAT-3′, *Gapdh* reverse 5′-CCTTCCACAATGCCAAAGTT-3′; *Il1b* forward 5’TGACAGTGATGATGAGAATGACCTGTTC-3′; *Il1b reverse* 5′-TTGGAAGCAGCCCTTCATCT-3′. All primers were supplied by Sigma.

### Endogenous caspase-1 activity using FAM-YVAD–fluoromethyl ketone (FMK)

Peritoneal macrophages into coverslips or 96 well plate were treated with carrageenan were incubated 45 min with FAM-YVAD-FMK (1,5 ratio) (Immunochemistry Technologies; Cat. n. 98) as recommended by the manufacturer’s instructions. Cells were washed with sterile PBS at room temperature twice. The coverslips were inverted in assembly medium containing DAPI (DAPI Prolong Gold® antifade reagent, invitrogen) on the fluorescence slides for subsequent capture of the images, acquired on confocal microscope (Leica TCS SP5). Caspase-1 activity was also analyzed by flow cytometry (BD FACS Verse) considering FLICA-positive cells.

### ATP quantification

The supernatant from the carrageenan-stimulated cells was collected and the ATP quantification assay was immediately performed. The concentration of ATP was determined by bioluminescence according to ATP lite One-step kit recommendations (ATP Dosage Kit − 6,016,941, Perkin-Elmer, Waltham, MA, USA). The ATP lite assay system is based on the production of light elicited by the reaction of ATP with added luciferase and D-luciferin. ATP concentrations were determined using the standard curve for quantification of the samples.

### Western blotting

Cells were lysate in protein extraction buffer containing protease inhibitor, while the supernatants were prepared by precipitation of proteins using chloroform/methanol. IL-1β immature (pro-IL-1β; ~ 35 kDa) and active (~ 17 kDa) forms and Casp1 immature (~ 45 kDa) and active (~ 20 kDa) were analyzed in cell lysate and supernatant by western blotting. Proteins were separated by SDS-polyacrylamide gel electrophoresis (15% SDS-PAGE) and transferred to nitrocellulose membranes (Amersham Pharmacia Biotech – GE10600002). Membranes were incubated for 2 h at room temperature with blocking buffer, followed by incubation with indicated primary antibodies: goat antibody to IL-1β p35/p-17 (1:200, Sigma Aldrich, cat. n. I3767), rat monoclonal antibody to caspase-1 p45/p20 (1:400, Genentech, 4B4), goat antibody to ASC (p25) (1:500, Santa Cruz Biotechnology, SC- Cat. N° 33,958), rabbit antibody to phospo-Syk (p72) (11,000, Invitrogen, MA5–14918), rabbit antibody to Total Syk p72 (11,000, Invitrogen, PA5–17812), mouse anti-β-Actin (11,000, C4, Santa Cruz sc-47,778). The enhanced chemiluminescence luminol reagent (Immobilon Forte Western HRP Substrate - WBLUF0500, Millipore) was used for antibody detection as described in the instruction manual. Membranes were analyzed by using an imaging system Chemic Doc TM XRS + System (Bio-Rad Laboratories) to measure the intensity of the optical density of each band.

### Quantification of ASC specks formation

Cells were collected from 8 to 10-week-old transgenic ASC-mCitrine mice by flushing and retrieving RPMI from the peritoneal cavity. After washing, cell density was adjusted and 2 × 10^5^ cells were transferred to an 8-well chamber and incubated overnight at 37 °C + 5% CO 2. Afterward, non-adherent cells were removed. Stimulation was performed in fresh medium with Cg (300 μg/mL) for 24 h in fresh RPMI medium.

### Confocal microscopy and ASC-specking analysis

Briefly, cells were fixed with 4% PFA and then washed with PBS. Cells from Transgenic ASC-mCitrine mice nuclear staining was performed with Hoechst (33528–3 μM). Representative images were acquired using Leica TCS SP5 confocal system. For ASC speck counting, the software Cell Profiler 3.1.5 was used. Nuclei and ASC specks were identified by specific threshold strategies, considering the size and signal intensity. The proportion of ASC specks/nuclei was expressed in percentage.

### Cg-induced colitis and samples collection

The animals were gently restrained to immobilize the head. In the vertical position a stainless steel bulb tipped gavage needle is attached to a syringe and used to deliver the Cg (5%) directly into the stomach. The volume administered was 0.2 ml per mice during 20 days. The body weight change was evaluated every morning before gavage and quality of stool were recorded each day. Upon completion of the protocol, all animals were used for colonoscopy examination and then sacrificed. The proximal and sigmoid colon regions were removed to procedure the histopathological and molecular analysis.

### Colonoscopy examination

The protocol and images presented in this study were prepared with a flexible endoscope (Karl storz GmbH & Co. KG Tele Pack VET X). The endoscopy system was set up before anesthetizing the animals, following the manufacturer’s instructions. A video ureteroscope was used for this protocol. Previously a colon lavage with saline was performed to remove residual feces. The endoscope was inserted and recording started for imaging, where necessary air was injected with a syringe to slowly separate the intestinal walls. As the camera in progress the splenic flexure region, descending colon and rectum were examined. Spontaneous bleeding in the rectum and colon (defined as natural mucosal bleeding not associated with traumatic endoscopy), transparency of the colon wall (transparency was defined as the ability to visualize the intramural blood vessels in the surrounding viscera) and the number of focal lesions (Edematous areas, erosions, ulcers). Inflammatory characteristics were recorded using the colonic inflammation score of severity with decimal identifiers.

### Histological analysis and myeloperoxidase (MPO) immunostaining

Paraffin-embedded tissue sections were cut into 4-mm-thick sections and stained with hematoxylin-eosin for the histopathological examination. Inflammatory cell infiltrate into the distal colon wall was graded according to the following four-point scale: 0, none damage; 1, mild damage; 2, moderate damage; 3, severe damage. Infiltration of neutrophils was also estimated by means of the immunohistochemical assay for myeloperoxidase. Paraffin-embedded colon cross-sections (4-mm-thick) mounted on poly-L-lysine-coated slides were deparaffinized, rehydrated, immersed in 10 mmol/L citrate buffer, pH 6.0, and submitted to heat-induced epitope retrieval using a vapor lock for 40 min. After heating, the slides were allowed to cool to room temperature and were briefly washed with a Tris-buffered saline solution. Endogenous peroxidase activity was blocked with 3% hydrogen peroxide for 15 min. A protein block solution (Spring Bioscience, Pleasanton, CA, U.S.A.) was used to block nonspecific binding and incubated with the slides for 10 min. Immunohistochemical staining was performed using a biotin-free polyvalent horseradish peroxidase (HRP) system (Reveal kit, Spring Bioscience). The intestinal sections were then incubated with the rabbit monoclonal anti-MPO antibody (clone EPR20257, Abcam, Cambridge, United Kingdom) as the primary antibody, diluted 1:1000, for 1 h at room temperature (approximately 25 °C) in a humidified chamber. After washing with phosphate-buffered saline, a secondary antibody (Reveal Kit Complement, Spring Bioscience) was applied for 10 min. The sections were incubated with an HRP-conjugated secondary antibody (Reveal Kit HRP Conjugate, Spring Bioscience) for 15 min and developed with 3. 3-diaminobenzidine tetrahydrochloride (Spring Bioscience) in phosphate-buffered saline, pH 7.5, for 5 min. Light Mayer’s hematoxylin was applied as a counterstain. The slides were then dehydrated in a series of ethanol dilutions and mounted with Permount (Fischer, Fairlawn, NJ, U.S.A.). The numbers of myeloperoxidase-positive leukocytes were counted in three separate cross-sections of distal colon for each mouse. The results are expressed in neutrophil number per cross-section.

### MPO activity assay

Neutrophil migration was assessed in the distal colon tissues of mice using a myeloperoxidase (MPO) kinetic colorimetric assay as previously described [12]. After 20 days Cg-treatment, distal colon tissue were collected in 50 mM K2HPO4 buffer (pH 6.0) containing 0.5% hexadecyltrimethylammonium bromide and kept at 280 °C until use. The tissue were homogenized using a Polytron (PT3100) and centrifuged at 16,100 g for 4 min. The resulting supernatant was assayed for MPO activity spectrophotometrically at 450 nm (SpectraMax, Molecular Devices, San Francisco, CA), with 3 readings in 1 min. The MPO activity of samples was compared with a standard curve of neutrophils. Briefly, 10 mL of the sample was mixed with 200 mL of 50 mM phosphate buffer, pH 6.0, containing 0.167 mg/mL o-dianisidine dihydrochloride and 0.0005% hydrogen peroxide. The results are presented as MPO activity (number of neutrophils per milligram of tissue).

### Statistical analysis

Statistical analysis was performed by one-way ANOVA followed by a Holm-Sidak’s post hoc test and the unpaired t-test to determine the level of statistical significance. Data are expressed as mean (SD). All western blotting image were representative of at least three independent experiments. For colitis colonoscopy score, comparison of groups was performed using appropriate non-parametric tests (Mann–Whitney U test, analysis of variance, Kruskal–Wallis test). Differences were considered statistically significant when the *p* <  0.05 (*p <  0.05; **p <  0.01; and ***p <  0.001; ns, not significant). Data were plotted and analyzed with GraphPad Prism 6.0 software (GraphPad, San Diego, California, USA).

## Results

### Cg differentially requires TLR4 downstream signaling to secrete IL-1β and TNF by peritoneal macrophages

**Fig. 1:**
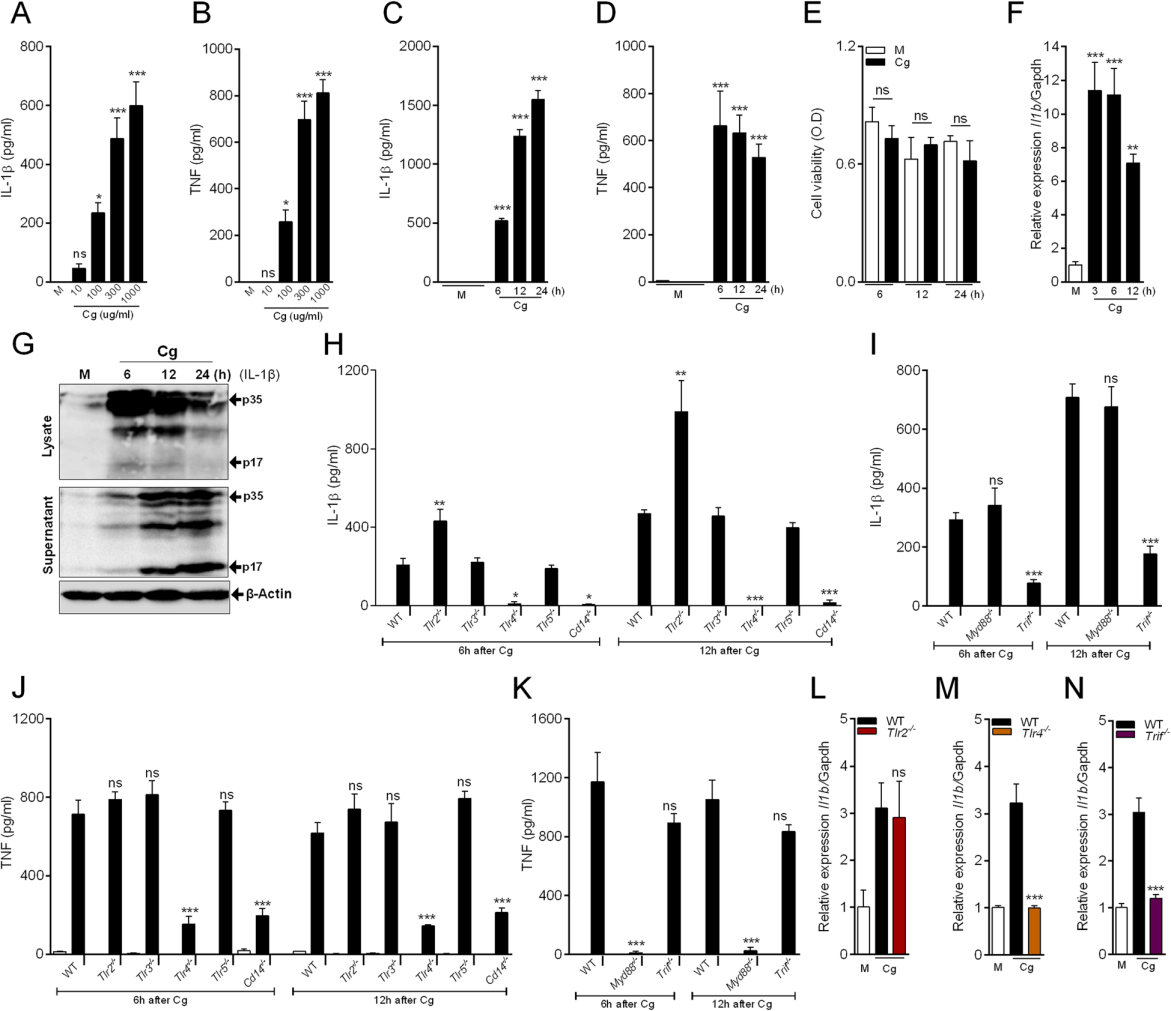
Cg induces cytokines (TNF and IL-1β) production/release by peritoneal macrophages: role of TLR4 signaling pathway. **a, b** Peritoneal macrophages were stimulated with different concentration of Cg (30-1000 μg/ml). After 6 h, supernatants were collected for quantification of IL-1β and TNF by Elisa. **c, d** Peritoneal macrophages were stimulated with Cg (300 μg/ml). After indicated times, supernatants were collected for quantification of IL-1β and TNF by Elisa. **e** Cell viability were analysis by MTT assay at indicated times after Cg (300 μg/ml) stimulation. **f** Peritoneal macrophages were stimulated with Cg (300 μg/ml). After indicated times, *Il1b* mRNA gene expression was determined by qPCR **(g)** Representative western blotting for IL-1β expression (immature form; p35 and active form; p17) in the supernatant and cell lysate from Cg (300 μg/ml)-stimulated macrophages. Beta-actin was used as a loading control. **h, i, j, k** Peritoneal macrophages harvested from naive WT, *Tlr2*^*−/−*^*, Tlr3*^*−/−*^*, Tlr4*^*−/−*^*, Tlr5*^*−/−*^*, Cd14*^*−/−*^*, Myd88*^*−/−*^ and *Trif*^*−/−*^ mice were stimulated with Cg (300 μg/ml) or medium. At indicated time points, the supernatants were collected for quantification of IL-1β and TNF by Elisa. **l, m, n** Peritoneal macrophages harvested from naive WT, *Tlr2*^*−/−*^*, Tlr4*^*−/−*^ and *Trif*^*−/−*^ were stimulated with Cg (300 μg/ml) or medium. After 3 h, the *Il1b* mRNA expression was determined by qPCR. Data are represent the mean ± SD of four independent experiments compared Control vs WT (Cg) or WT vs Knockout groups to determine the level of statistical significance (*p < 0.05; **p < 0.01; and ***p < 0.001; ns, not significant)

To study the molecular basis of Cg-induced macrophages production/release of IL-1β and TNF, firstly we characterize our in vitro model. For that, primary culture of peritoneal macrophages from naïve C57BL/6 (WT) mice was used and Cg induced a significant, concentration-dependent release of IL-1β and TNF at 6 h (Fig. 1a, b). The concentration of 300 μg/ml of Cg induced the peak of IL-1β release and was chosen as the concentration for the next experiments. The time-course of measurement revealed that IL-1β release increased progressively from 6 h, peaking between 12 and 24 h after Cg stimulation (Fig. 1c). TNF release was also observed in the supernatant of Cg-stimulated macrophages (Fig. 1d), although the time-course shows the peak is already reached at 6 h after Cg incubation (Fig. 1d) in the absence of cytotoxicity (Fig. 1e). Given that the release of biologically active IL-1β require, at least, two important steps/signals, one for the formation of mRNA for pro-IL-1β (*Il1b*) and a second step, the processing of pro-IL-1β (p35, ~ 35 kDa) into a mature form (p17, ~ 17 kDa), we next analyzed these two forms of IL-1β in peritoneal macrophages after Cg stimulation. Cg-stimulated macrophage increased *Il1b* mRNA expression, which attains the maximum after 3 h post stimulation decreasing thereafter (Fig. 1f). In addition, western-blotting analyses of cell lysate and supernatant from Cg-stimulated macrophages revealed a time- and compartment-dependent increase in pro-IL-1β (p35) and active IL-1β (p17) (Fig. 1g), suggesting that Cg alone induces the production and release of both forms of IL-1β by peritoneal macrophages.

We next sought to determine the molecular mechanisms underlying Cg-induced IL-1β by peritoneal macrophages. Initially, we evaluated the participation of TLRs (TLR2, TLR3, TLR4 and TLR5), their downstream signaling molecules (MyD88 and TRIF), as well as the CD14 co-receptor. The release of IL-1β was abrogated in Cg-stimulated peritoneal macrophages from *Tlr4*^*−/−*^ and *Cd14*^*−/−*^ mice compared to the control group (Fig. 1h), which was also observed for TNF release (Fig. 1j). Macrophages from *Tlr3*^*−/−*^ and *Tlr5*^*−/−*^ mice released similar amounts of both cytokines. On the other hand, the release of IL-1β, but not of TNF, were higher in macrophages from *Tlr2*^*−/−*^ mice (Fig. 1h and j). Surprisingly, the production of TNF (Fig. 1k) was abrogated in Cg-stimulated macrophages from *Myd88*^*−/−*^ mice, whereas the production of IL-1β was inhibited in macrophages from *Trif*^*−/−*^ mice but did not change in macrophages from *Myd88*^−/−^ mice (Fig. 1i). Importantly, Cg-stimulated peritoneal macrophages increase of *Il1b* mRNA expression is not observed in cells from *Tlr4*^*−/−*^ and *Trif*^*−/−*^ mice, although it was similar to control group in macrophages from *Tlr2*^*−/−*^ mice (Fig. 1l-n). To ensure that this TLR4-mediated effect is not due to Cg contamination with endotoxin (e.g. LPS), limulus amebocyte lysate test (LAL) was performed. The endotoxin level at Cg (300 μg/ml) concentration was below the detection limit. Lipopolysaccharide (LPS; 0.01–1000 ng/ml) was used as a positive control (Table 1). In addition, differently from we have observed with Cg, LPS alone was not able to induce the production of IL-1β by peritoneal macrophages, whereas both induced the production of TNF (Additional file 1). These results indicate that in response to Cg, peritoneal macrophages are able to produce and release the pro-inflammatory cytokine IL-1β, which, at least the mRNA for this cytokine depends on an unexpected TLR4/TRIF signaling pathway. Conversely, Cg-induced TNF release by peritoneal macrophages depends on the TLR4/MyD88 signaling. Noteworthy, TLR2-dependent signaling seems to counteract post-translational mechanisms involved in the production/release of IL-1β by peritoneal macrophages stimulated with Cg.

**Table 1:** LAL test in Cg solutions

Samples	Presence of endotoxin	Sensitivity	Gelation occurs	Endotoxin units (EU/ml)
Culture Medium	–	< 0,015	–	~ 1000
PBS solution	–	< 0,015	–	~ 1000
LPS 0,01 ng/ml	+	> 0,015	+	~ 6000
LPS 0,1 ng/ml	+	> 0,015	+	~ 12,000
LPS 1 ng/ml	+	> 0,015	+	~ 12,000
LPS 10 ng/ml	+	> 0,015	+	~ 24,000
LPS 100 ng/ml	+	> 0,015	+	~ 24,000
LPS 1000 ng/ml	+	> 0,015	+	~ 24,000
Cg 30 μg/ml	–	< 0,015	–	~ 1000
Cg 100 μg/ml	–	< 0,015	–	~ 1000
Cg 300 μg/ml	–	< 0,015	–	~ 1000

(+) Positive or (−) Negative detection for endotoxin presence

*Cg* Carrageenan, *LPS* Lipopolysaccharide, *EU/ml* corresponding endotoxin concentration

### Syk/ROS downstream of TRIF signaling are involved in *Il1b* mRNA expression by Cg-stimulated macrophages

**Fig. 2:**
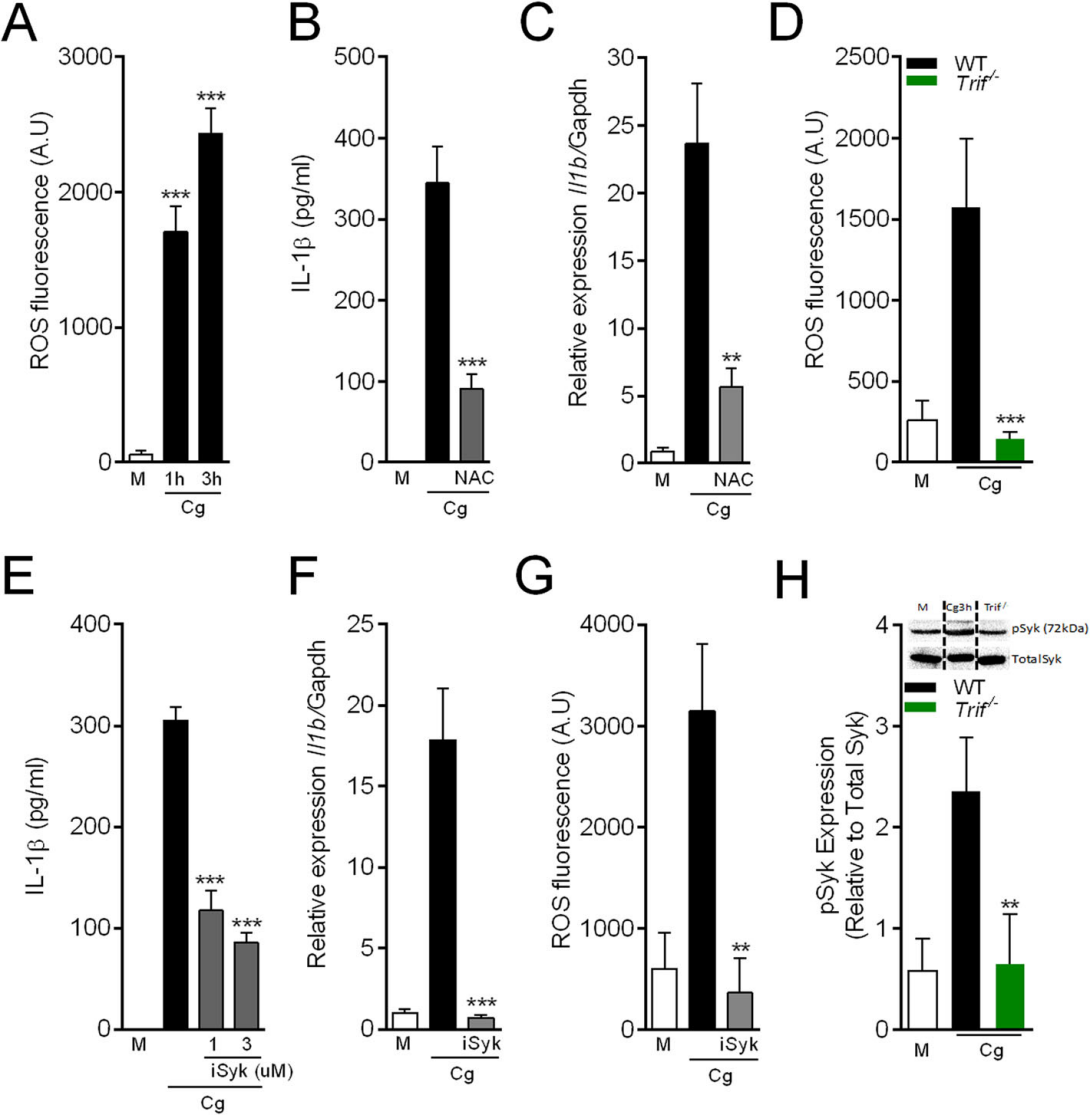
Role of TRIF/Syk/reactive oxygen species on Cg-induced *Il1b* mRNA. Peritoneal macrophages were stimulated with Cg (300 μg/ml) or medium. **a** ROS production were quantified in supernatant by the fluorescent probe assay (H2DCFDA) after indicated times. **b, c** The supernatant were collect after pre-treated cells with N-acetylcysteine (NAC – 3 mM) to quantify *Il1b* mRNA gene expression by qPCR and IL-1β production by Elisa after Cg (6 h). **d** Quantification of ROS production by (H2DCFDA probe) in cells from naive WT vs deficient for *Trif*^*−/−*^ after Cg (3 h). **e, f** Pre-treated cells with selective inhibitor of Syk (iSyk 1, 3 μM) and stimulated with Cg (6 h) to quantify IL-1β by ELISA and *Il1b* mRNA gene expression induced by Cg (3 h), medium or (iSyk 3 μM). **g** Quantification of ROS production (H2DCFDA probe) stimulated with Cg and iSyk (3 uM) after 3 h. **h** Western blotting analysis of (pSyk) expression compared cells from WT vs *Trif*^*−/−*^. Data are represent the mean ± SD of four independent experiments compared Control vs WT (Cg) or WT vs Knockout/Treatments groups to determine the level of statistical significance (*p < 0.05; **p < 0.01; and ***p < 0.001; ns, not significant)

It attempt to further understand the molecular mechanisms involved in the production of IL-1β by Cg-stimulated macrophages, next, we sought to find possible mechanisms downstream to TRIF pathway. There is recent evidence suggesting reactive oxygen species (ROS) as an important driver for *Il1b* mRNA as downstream signaling of TLRs pathway [28]. Testing this hypothesis, firstly we found that peritoneal macrophages stimulated with Cg increased the production of ROS (Fig. 2a). Furthermore, pretreatment of macrophages with the antioxidant, N-acetylcysteine (NAC) inhibited both Cg-induced IL-1β release and the up-regulation of its mRNA expression (Fig. 2b and c), without affecting the release of TNF (Additional file 2). Connecting Cg-induced TRIF signaling with ROS production in peritoneal macrophages, it was found that Cg-induced ROS production was not observed in macrophages from *Trif*^*−/−*^ mice (Fig. 2d). These results indicate that TRIF/ROS signaling mediates the induction of *Il1b* mRNA in Cg-stimulated macrophages.

An important signaling pathway in macrophages responsible for ROS production is the spleen tyrosine kinase (Syk) [29]. Syk activation is upstream signaling in the production of ROS stimulated by a range of different stimuli [30–33]. Therefore, we next evaluated whether Syk pathway would be involved in the production of ROS by Cg-stimulated macrophages and consequently to drive *Il1b* mRNA. We found that pharmacological inhibition of Syk (iSyk) reduced the release of IL-1β in Cg-stimulated macrophages, which was associated with an inhibition of the expression of *Il1b* mRNA (Fig. 2e and f), but does not affect TNF release (Additional file 2). Moreover, the Syk inhibitor abrogates the production of ROS in Cg-stimulated macrophages (Fig. 2g). Finally, the activation of Syk (increase in pSyk) pathway in Cg-stimulated macrophages was not observed in *Trif*^*−/−*^ macrophages (Fig. 2h). Collectively these data indicate that the induction of *Il1b* mRNA in Cg-stimulated peritoneal macrophages depends on a TLR4/TRIF/Syk/ROS pathway.

### NLRP3/ASC/Casp1 inflammasome activation is required for processing of IL-1β in macrophages stimulated with Cg

**Fig. 3:**
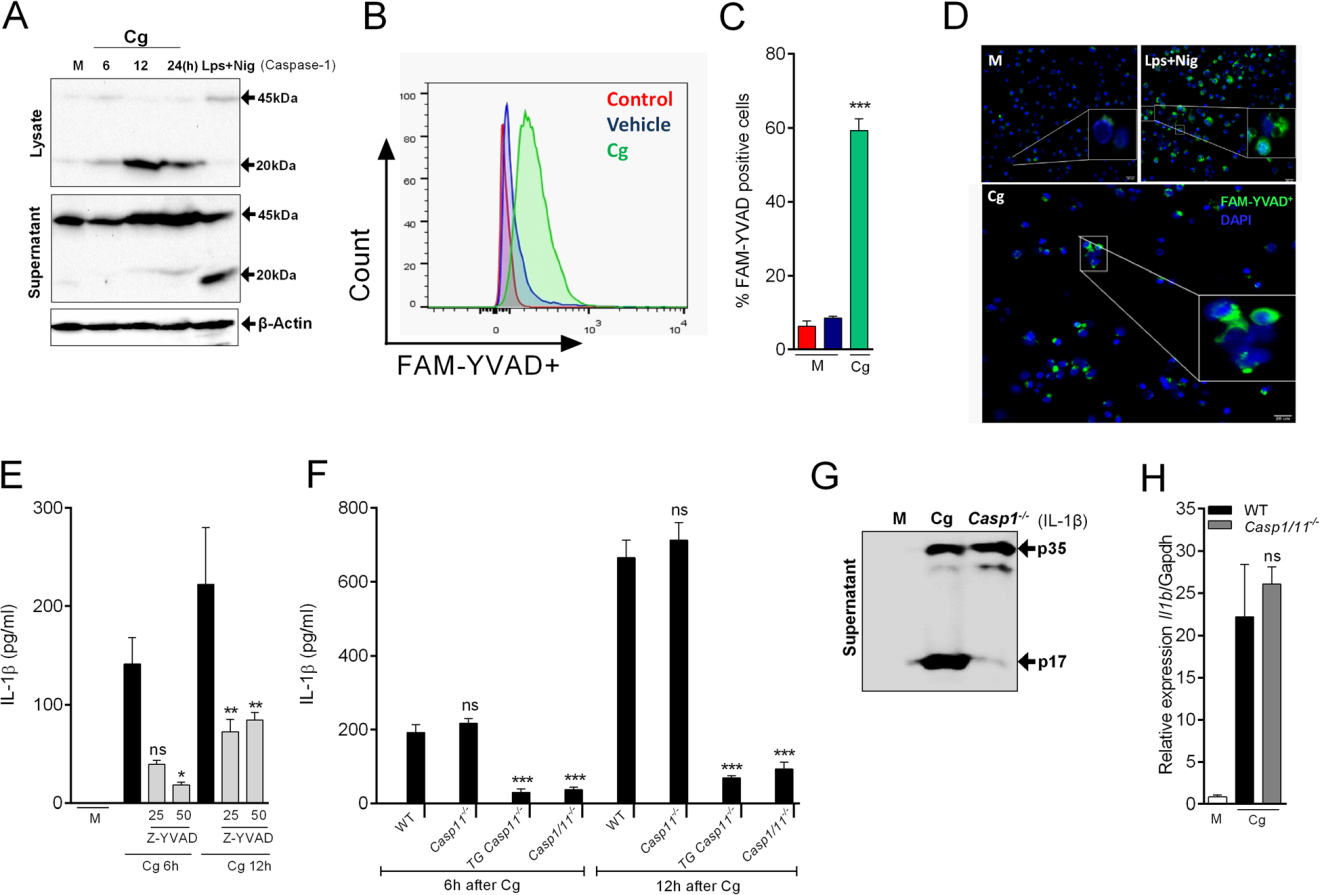
Casp1, but not Casp11, mediates IL-1β production by Cg-stimulated macrophages. Peritoneal macrophages were stimulated with Cg (300 μg/ml) or medium. **a** Representative western blotting for immature (p45) and active form (p20) of Caspase-1 at indicated times after Cg incubation. Beta-actin was used as a loading control. **b, c** Representative histogram and quantification of FAM-YVAD^+^ cells after Cg incubation (12 h). **d** Representative images of caspase-1 activity (FAM-YVAD^+^; green staining) after 12 h of Cg stimulation. Cell nucleus were stained with DAPI (blue). LPS primed macrophages plus nigericin was used as a positive control. **e** Cells were pre-incubated with a selective inhibitor of Caspase-1 (Z-YVAD) (25, 50 μM - 30 min) and then stimulated with Cg. After indicated times, the supernatants were collected for IL-1β quantification by Elisa. **f** Peritoneal macrophages harvested from naive WT, *Casp11*^*−/−*^, *Casp1/11*^*−/−*^, or *Casp1*^*−/−*^*Casp11Tg* mice were stimulated with Cg or medium. At indicated time points, the supernatants were collected for quantification of IL-1β by Elisa. **g** Representative western blotting expression of immature form (p35) and the active form (p17) of IL-1β WT vs *Casp1/11*^*−/−*^ after Cg (24 h). **h** Peritoneal macrophages harvested from naive WT or *Casp1/11*^*−/−*^ mice were stimulated with Cg or medium. After 3 h, the *Il1b* mRNA expression was determined by qPCR. Data are represent the mean ± SD of four independent experiments compared Control vs WT (Cg) or WT vs Knockout/Treatments groups to determine the level of statistical significance (*p < 0.05; **p < 0.01; and ***p < 0.001; ns, not significant)

Casp1 is the major enzyme regulating the processing of pro-IL-1β into mature IL-1β in macrophages [34]. Therefore, we tested whether Casp1 is involved in the production/release of IL-1β by peritoneal macrophages stimulated with Cg. Firstly, we analyzed whether Cg activates Casp1 in peritoneal macrophages as detected by cleaved form (p20). We performed western blotting analysis and detected the cleaved form of Casp1 (p20) in the lysates and supernatant of Cg-stimulated macrophages (Fig. 3a). Nigericin stimulated LPS-primed macrophages was used as a positive control (Fig. 3a). Corroborating with these data, we incubated Cg-stimulated macrophages with FAM-FLICA-YVAD, a fluorescent dye that selectively binds to active Casp1. FACS analysis showed that Cg induced significant Casp1 activation in macrophages (Fig. 3b and c). Increased FAM-YVAD^+^ positive macrophages after Cg stimulation can also be visualized by fluorescent microscopy (Fig. 3d). Since Cg induced the activation of Casp1 in macrophages, we determined its involvement in IL-1β processing. Cg-stimulated C57BL/6 macrophages release IL-1β, which was inhibited by a Casp1 selective inhibitor (Fig. 3e) and IL-1β was abrogated in Cg-stimulated macrophages isolated from *Casp1/11*^−/−^ mice (Fig. 3f), whereas both pro-IL1β (p35) and *Il1b* mRNA gene expression was similar to control group (Fig. 3g and h). Excluding the participation of Casp11 for this process, the release of IL-1β by peritoneal macrophages isolated from *Casp11*^−/−^ mice and stimulated with Cg was similar to WT controls, whereas it still inhibited in macrophages from *Casp1*^−/−^*Casp11 Tg* mice (Fig. 3f). Noteworthy, none of these mouse genotypes or pharmacological treatments changed the ability of Cg to stimulate the production of TNF by peritoneal macrophages (Additional file 3). These data indicate that Cg induces production/release of IL-1β by peritoneal macrophages depends on canonical Casp1 activation, but not of Casp11.

**Fig. 4:**
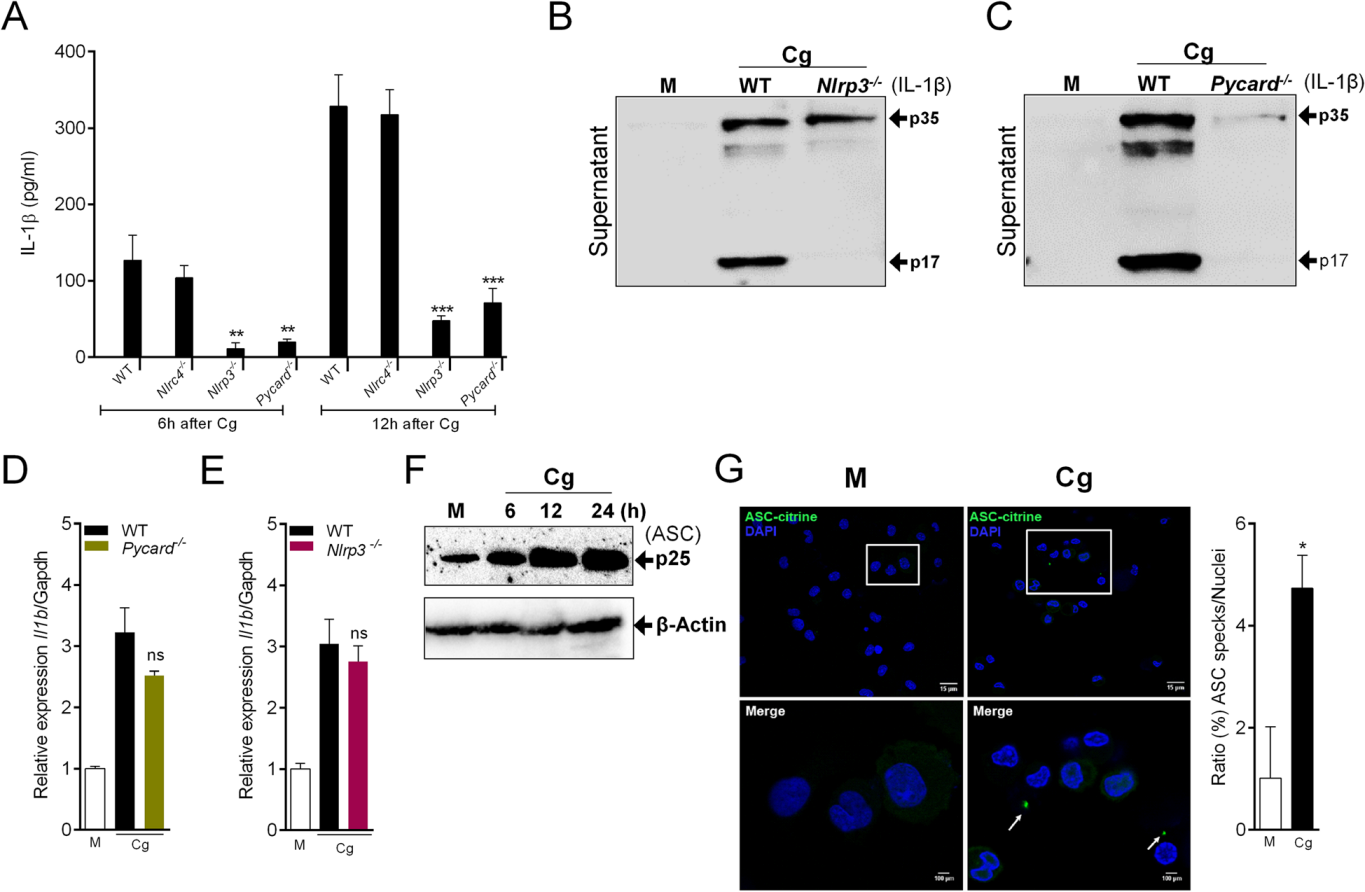
NLRP3 Inflammasome is required for IL-1β production by Cg-stimulated macrophages. **a** Peritoneal macrophages harvested from naive WT, *Nlrp3*^*−/−*^*, Nlrc4*^*−/−*^*, Pycard*^*−/−*^ mice were stimulated with Cg (300 μg/ml) or medium. At indicated time points, the supernatants were collected for quantification of IL-1β by Elisa. **b, c** Representative western blotting for immature (p35) or active form (p17) of IL-1β in the supernatant of Cg-stimulated macrophages (24 h). Macrophages were harvested from WT, *Nlrp3*^*−/−*^ *or Pycard*^*−/−*^ mice. **d, e** Peritoneal macrophages harvested from naive WT, *Nlrp3*^*−/−*^ *or Pycard*^*−/−*^ mice were stimulated with Cg (300 μg/ml) or medium. After 3 h, the *Il1b* mRNA expression was determined by qPCR. **f** Representative western blotting for ASC (p25) expression in Cg- or medium-stimulated macrophages harvested from WT mice. **g** Representative images (confocal) and quantification of ASC specks (green dots) formation in Cg (24 h)-stimulated peritoneal macrophages harvested from ASC-mCitrine reporter mice. Cell nucleus were stained by DAPI (blue). Data are represent the mean ± SD of four independent experiments compared Control vs WT (Cg) or WT vs Knockout groups to determine the level of statistical significance (*p < 0.05; **p < 0.01; and ***p < 0.001; ns, not significant)

Casp1 activation and IL-1β processing are generally preceded by the assembling of inflammasome platforms, in which NLRP3 and NLRC4 inflammasomes are the most studied [35–37]. We investigated whether the production/release of IL-1β by Cg-stimulated macrophages depends on one of these inflammasome platforms. We found that the release of IL-1β by Cg-stimulated peritoneal macrophages was not observed in macrophages from *Nlrp3*^−/−^ and *Pycard/Asc*^−/−^ mice (Fig. 4a). On the other hand, no difference was observed with macrophages from *Nlrc4*^−/−^ mice (Fig. 4a). The data were confirmed by western blotting analyses which showed that the levels of mature IL-1β (p17) in the supernatant of Cg-stimulated macrophages was lower in *Nlrp3*^*−/−*^ and *Pycard*^*−/−*^ macrophages compared C57BL/6 controls (Fig. 4b and c). Nevertheless, TNF release (Additional file 3) and *Il1b* mRNA remain unchanged in Cg-stimulated macrophages from these different genotypes (Fig. 4d and e). Supporting, the role of NLRP3/ASC inflammasome for the processing of IL-1β, we found that ASC expression was up-regulated in Cg-stimulated peritoneal macrophages (Fig. 4f). Given that inflammasome assembly can be detected by the visualization of a dot-like structure called ASC speck [38], we performed fluorescence assay using peritoneal macrophages from ASC-mCitrine Tg reporter mice [26] stimulated with Cg. Notably, we revealed an increase in ASC speck formation at 24 h of Cg stimulation compared to control group (Fig. 4g).

### Mechanisms of NLRP3 inflammasome activation by cg in macrophages

**Fig. 5:**
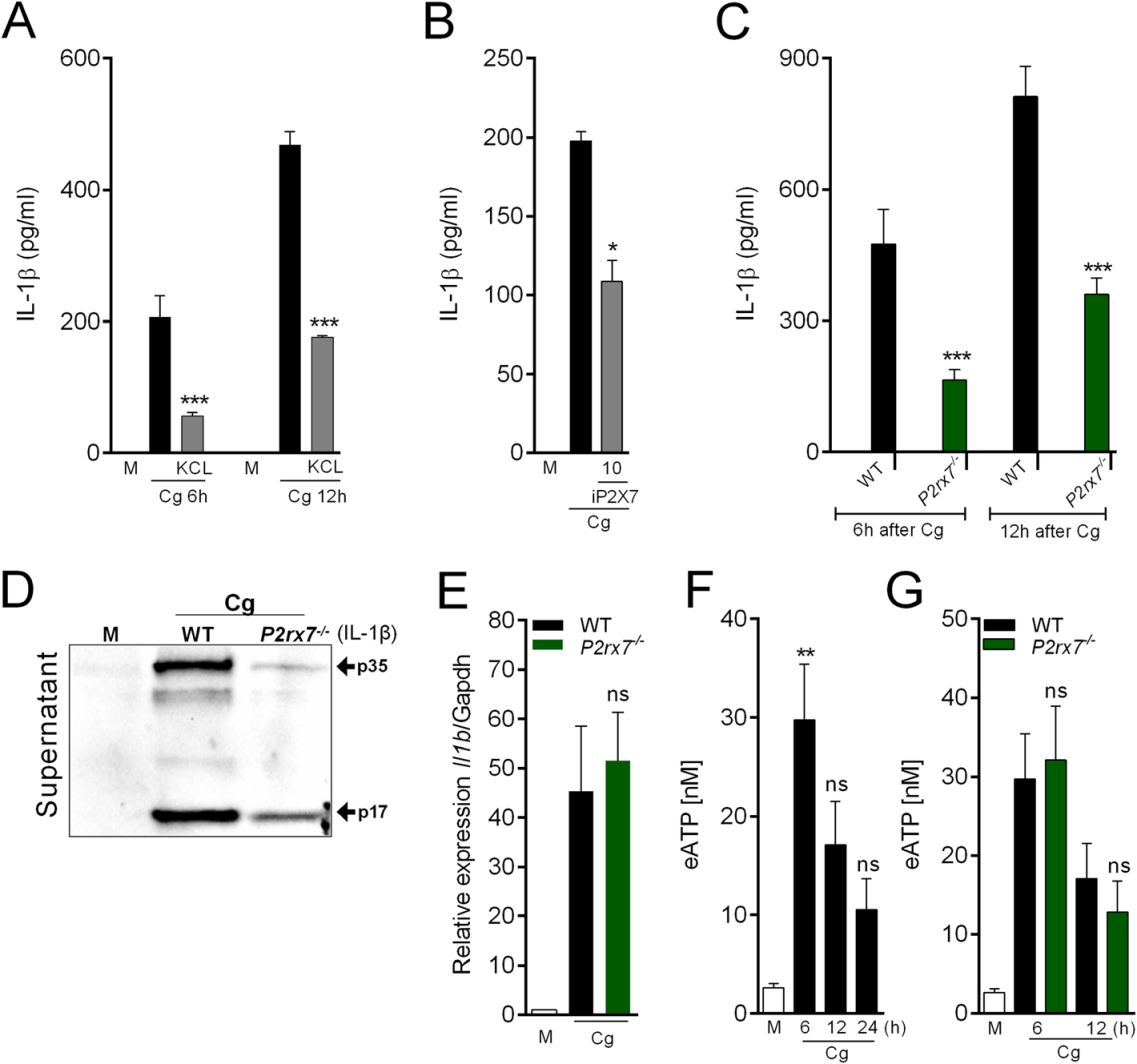
Role of eATP/P2X7R in the production of IL-1β by Cg-stimulated macrophages. **a** Peritoneal macrophages were maintained in regular medium or high-concentrated KCl (25 mM) medium and then stimulated by Cg (300 μg/ml). At indicated time points, the supernatants were collected for quantification of IL-1β by Elisa. **b** Peritoneal macrophages were pre-incubated with a selective inhibitor of P2X7R (iP2x7) (10 uM - 30 min) and then stimulated with Cg (300 μg/ml). After 6 h, the supernatants were collected for IL-1β quantification by Elisa. **c** Peritoneal macrophages harvested from naive WT or *P2xr7*^*−/−*^ mice were stimulated with Cg or medium. At indicated time points, the supernatants were collected for quantification of IL-1β by Elisa. **d** Representative western blotting for immature (p35) or active form (p17) of IL-1β in the supernatant of Cg-stimulated macrophages (24 h). Macrophages were harvested from WT or *P2rx7*^*−/−*^ mice. **e** Peritoneal macrophages harvested from naive WT or *P2rx7*^*−/−*^ mice were stimulated with Cg (300 μg/ml) or medium. After 3 h, the *Il-1β* mRNA expression was determined by qPCR. **f** Peritoneal macrophages harvested from naive WT mice were stimulated with Cg. At indicated times, the supernatants were collected for quantification of eATP concentration. **g** Peritoneal macrophages harvested from naive WT or *P2xr7*^*−/−*^ mice were stimulated with Cg or medium. At indicated times, the supernatants were collected for quantification of eATP concentration. Data are represent the mean ± SD of four independent experiments compared Control vs WT (Cg) or WT vs Knockout/Treatments groups to determine the level of statistical significance (*p < 0.05; **p < 0.01; and ***p < 0.001; ns, not significant)

Several intracellular mechanisms are involved in NLRP3 inflammasome activation [39–41]. Among them, potassium efflux triggers NLRP3 inflammasome activation [42]. We found that high extracellular K^+^ levels abrogated Cg-induced IL-1β released by macrophages (Fig. 5a), without affecting TNF release (Additional file 4), suggesting that canonical upstream mechanisms of NLRP3 are involved in the release of IL-1β by Cg-stimulated macrophages. On the other hand, to check deeply the NLRP3 mechanism we found that Cg don’t acts by changing osmolarity (Additional file 5) [43].

Extracellular ATP (eATP) acting through the purinergic receptor P2X7 (P2X7R) triggers potassium efflux in macrophages that activates NLRP3 inflammasome [44]. Therefore, the connection between P2X7R pathway for Cg-induced NLRP3 inflammasome activation and IL-1β processing was tested. Corroborating this hypothesis, it was found that Cg-induced IL-1β produced by peritoneal macrophages was reduced either in cells treated with a selective pharmacological inhibitor of P2X7R (Fig. 5b) or in macrophages from *P2rx7*^*−/−*^ mice (Fig. 5c). The western blotting analysis also revealed a reduction in the levels of mature IL-1β in the supernatant of Cg-stimulated macrophages from *P2rx7*^*−/−*^ mice compared to control (Fig. 5d). However, neither the *Il1b* mRNA gene expression (Fig. 5e) nor the release of TNF (Additional file 6) in Cg-stimulated macrophages was changed. To support the participation of P2X7R in Cg-induced macrophage production of IL-1β, we measured the release of eATP from peritoneal macrophages stimulated with Cg. Indeed, there is an increase in eATP levels in Cg-stimulated macrophages, which is maximum at 6 h after stimulation (Fig. 5f). Notably, the increase of eATP levels in Cg-stimulated macrophages was similar in macrophages from *P2rx7*^*−/−*^ mice compared to the control group (Fig. 5g).

**Fig. 6:**
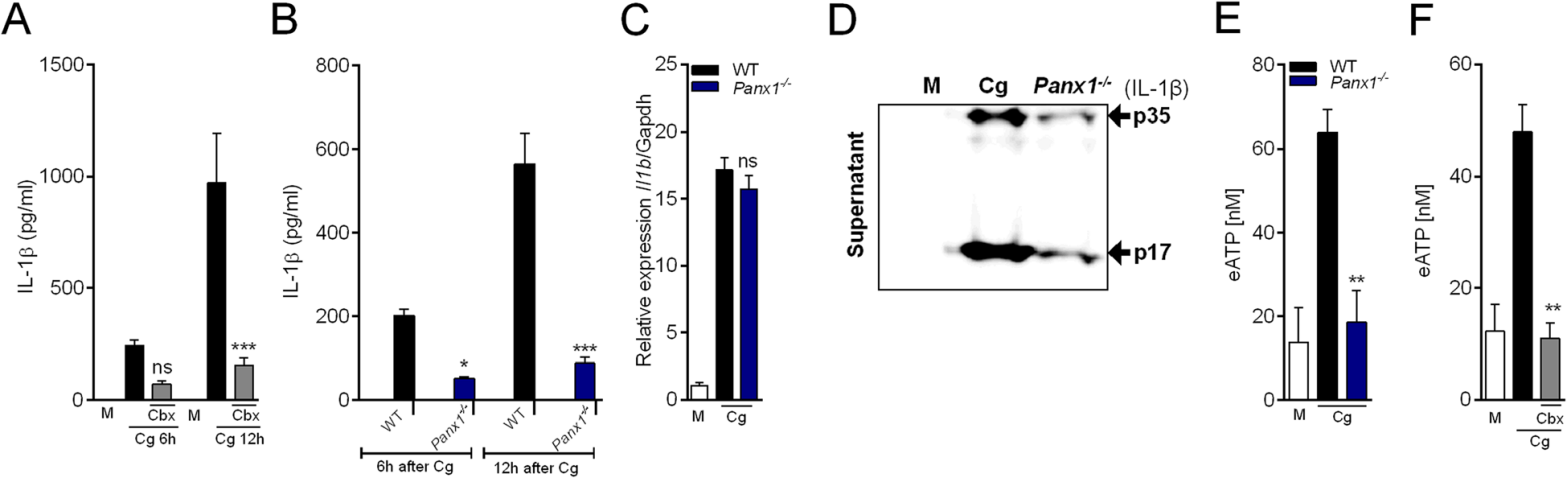
Cg-induced IL-1β production by macrophages depends on ATP efflux through Panx1 channel. **a** Peritoneal macrophages were pre-incubated with carbenoxolone (Cbx, 50 uM - 30 min) and then stimulated with Cg (300 μg/ml). At indicated times, the supernatants were collected for IL-1β quantification by Elisa. **b** Peritoneal macrophages harvested from naive WT or *Panx1*^*−/−*^ mice were stimulated with Cg or medium. At indicated time points, the supernatants were collected for quantification of IL-1β by Elisa. **c** Peritoneal macrophages harvested from naive WT or *Panx1*^*−/−*^ mice were stimulated with Cg or medium. After 3 h, the *Il1b* mRNA expression was determined by qPCR. **d** Representative western blotting for immature (p35) or active form (p17) of IL-1β in the supernatant of Cg-stimulated macrophages (24 h). Macrophages were harvested from WT or *Panx1*^*−/−*^ mice. **e** Peritoneal macrophages harvested from naive WT or *Panx1*^*−/−*^ mice were stimulated with Cg or medium. After 6 h, the supernatants were collected for quantification of eATP concentration. **f** Peritoneal macrophages were pre-incubated with carbenoxolone (Cbx, 50 uM - 30 min) and then stimulated with Cg. After 6 h, the supernatants were collected for quantification of eATP concentration. Data are represent the mean ± SD of four independent experiments compared WT vs Knockout/Treatments groups to determine the level of statistical significance (*p < 0.05; **p < 0.01; and ***p < 0.001; ns, not significant)

Pannexin-1 (Panx1), a large-pore channel, is a recognized mechanism by which intracellular ATP is released from cells [45, 46]. Once released via Panx1, ATP acts on P2X7R to amplify potassium ion efflux that in the last instance promotes NLRP3 inflammasome activation [39]. Thus, we investigated the involvement of Panx1 channels for Cg-induced IL-1β release by peritoneal macrophages. Non-selective pharmacological inhibition of Panx1 channels with carbenoxolone (Cbx), reduced IL-1β release from Cg-stimulated macrophages (Fig. 6a). These results were confirmed in Cg-stimulated macrophages from *Panx1*^*−/−*^ mice (Fig. 6b). Noteworthy, the increase in *Il1b* mRNA expression induced by Cg was similar in peritoneal macrophages from *Panx1*^*−/−*^ mice compared to the control group (Fig. 6c). Importantly, both pro-IL1β and IL-1β active form is reduced in macrophages supernatant from *Panx1*^*−/−*^ mice (Fig. 6d). The TNF release was not changed in Cg-stimulated macrophages from *Panx1*^*−/−*^ mice or in macrophages treated with Cbx (Additional file 6). Interestingly, the increase in eATP levels in Cg-stimulated macrophages was not observed in cells from *Panx1*^*−/−*^ mice or in cells treated with Cbx (Fig. 6e and f). Altogether, these results indicate that Panx1/eATP/P2X7R/potassium efflux pathway is upstream mechanisms involved in Cg-induced IL-1β production by peritoneal macrophages.

### Cg induced experimental colitis depends on NLRP3 inflammasome

**Fig. 7:**
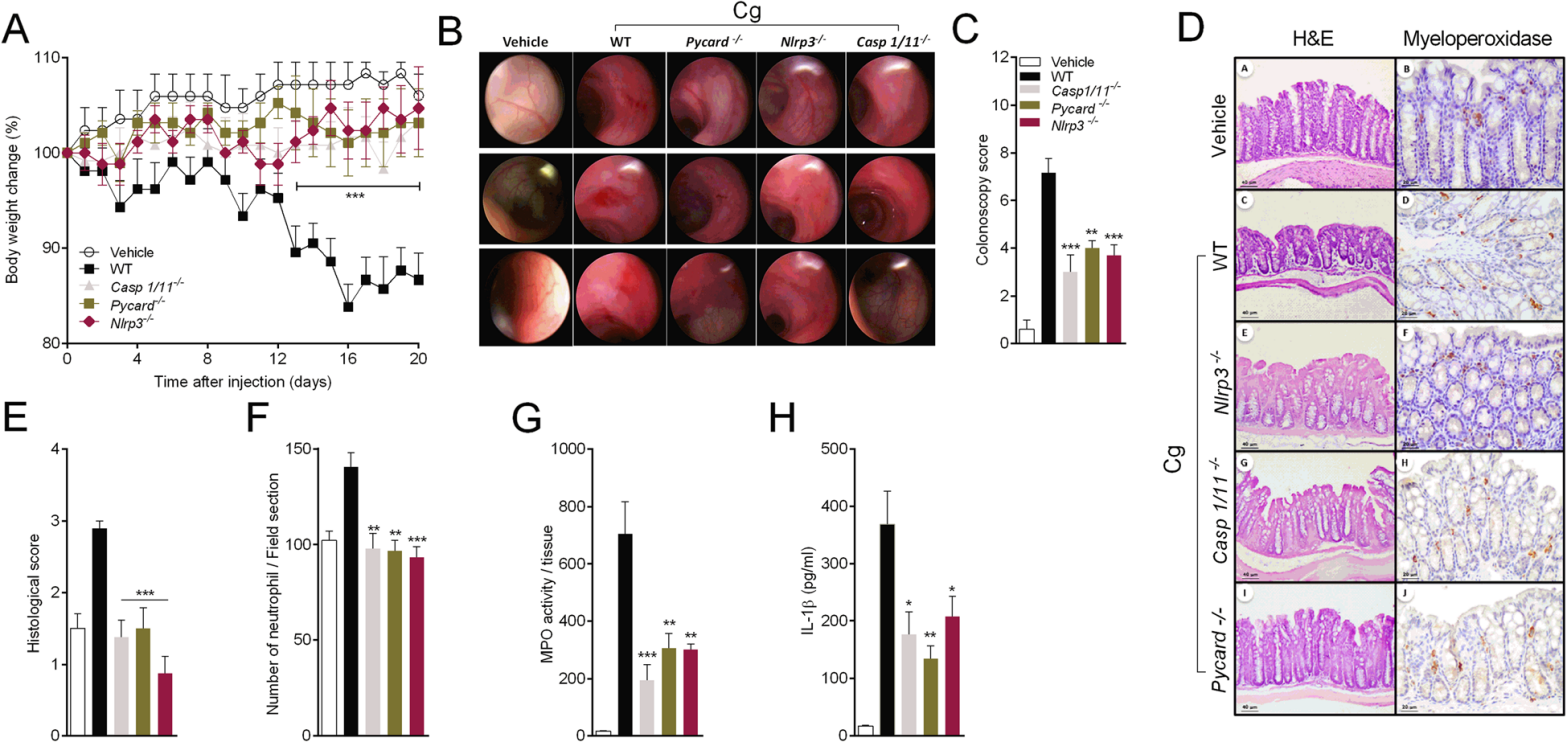
Cg-induced colitis in mice depends on NLRP3 inflammasome. The naive WT and *Nlrp3*^*−/−*^*, Casp1/11*^*−/−*^ and *Pycard*^*−/−*^ mice received Cg 5% or saline daily by gavage. **a** Body weight was measured daily up to 20 days in all groups. After 20 days of treatment, colonoscopy examination was performed. **b, c** Representative colonoscopy images from the splenic flexure region, descending colon, rectum and Colitis colonoscopy scores were determined comparing the control group, WT, *Casp1/11* ^*−/−*^*, Pycard* ^*−/−*^ and *Nlrp3*^*−/−*^ mice. **d** Representative H&E and immunohistochemistry stained for MPO+ cells from colon sections 20 days after Cg (5%) treatment. **e** Histological severity scores were determined comparing the control group, WT, *Casp1/11* ^*−/−*^*, Pycard* ^*−/−*^ and *Nlrp3*^*−/−*^ mice 20 days after Cg (5%) treatment. **f** Infiltration of neutrophils into the distal colon wall was estimated using myeloperoxidase immunostaining. **g** MPO activity was determined in the colon tissue from control group, WT, *Casp1/11* ^*−/−*^*, Pycard* ^*−/−*^ and *Nlrp3*^*−/−*^ mice 20 days after Cg (5%) treatment. **h** The colon samples were also used for the quantification of IL-1β contents by Elisa. Data are represent the mean ± SD. (*p < 0.05; **p < 0.01; and ***p < 0.001)

Finally, we tested the role of NLRP3 inflammasome in a model of Cg-induced colitis [7, 47], which has been widely used in different species, including rats, mice, rabbits and guinea pigs [48–50]. Here, C57Bl6 mice received daily gavage Cg (5%) to induce the experimental colitis [6, 51, 52]. C57BL/6 mice treated with Cg lost body weight progressively reaching the maximum between 16 and 20 days after treatment (Fig. 7a). By contrast, *Nlrp3*^*−/−*^*, Pycard*^*−/−*^ and *Casp1*^*−/−*^ mice were protected from Cg-treatment induced weight loss (Fig. 7a). At 20 day after Cg treatment, high-resolution live colonoscopy analyses revealed that *Nlrp3*^*−/−*^*, Pycard*^*−/−*^ and *Casp1/11*^*−/−*^ were protected from Cg-induced colitis (Fig. 7b-c). Histological analyses demonstrated increased number of infiltrated inflammatory cells, especially neutrophils in the crypt layer (Fig. 7d-f). The colitis severity score and neutrophils infiltration showed a significant reduction in *Nlrp3*^*−/−*^*, Pycard*^*−/−*^ and *Casp1/11*^*−/−*^ mice compared to C57BL/6 mice (Fig. 7d-f). Quantitative analysis of MPO activity was also reduced in samples from *Nlrp3*^*−/−*^*, Pycard*^*−/−*^ and *Casp1/11*^*−/−*^ mice compared to C57BL/6 mice (Fig. 7g). Finally, we found that the level of IL-1β was reduced in the colon of *Nlrp3*^*−/−*^*, Pycard*^*−/−*^ and *Casp1/11*^*−/−*^ mice compared to the control animals (Fig. 7h). These results indicate that NLRP3 inflammasome activation plays a crucial role in Cg-induced colitis in mice.

## Discussion

Cg is a linear sulfated polysaccharide extracted from seaweed. Winter and co-authors [53] observed that Cg induces foot oedema when injected in the rat’s hind paw. From this, Cg injection in the experimental animal into different tissues has been extensively used as a model of sterile inflammation in preclinical studies [9, 10, 54, 55]. However, the molecular mechanisms by which Cg induces inflammation is not completely elucidated. Here, we provided unraveled evidence showing that Cg-induced TNF production by macrophages depends on activation of TLR4/CD14/MyD88 signaling pathway. On the other hand, Cg-stimulated macrophages produce *Il1b* mRNA through TLR4/CD14/TRIF follow by Syk phosphorylation and ROS production. Furthermore, it was found that pro-IL-1β processing into mature IL-1β requires the activation of the NLRP3 inflammasome, which was triggered by Panx1/P2X7 receptor pathway. Finally, we demonstrated in vivo that NLRP3 inflammasome plays a crucial role in Cg-induced colitis in mice.

Several studies related to the development of antiinflammatory drugs were based on the use of Cg-induced inflammation as experimental models of inflammatory diseases [56, 57]. Since the proinflammatory action of Cg is mainly dependent on initial activation of macrophages that in turn produce pro-inflammatory cytokines, including TNF and IL-1β [17, 18, 58] the knowledge of the molecular mechanisms of Cg-activated macrophages might help in the discovery of novel targets for anti-inflammatory drug development. In this context, the role of TLR4 for the proinflammatory actions of Cg has been reported [18, 59]. We confirmed and extended this hypothesis showing that, besides TLR4, CD14, a TLR4 adapting molecule, is also involved in Cg-induced macrophage production of cytokines. Although these evidence strongly suggest that macrophages activation by Cg requires the TLR4 pathway, a recent study showed Cg did not activate TLR4-HEK293 reporter cells [60]. These controversial results might be derived from different cell subtypes (macrophages X HEK293) and concentrations of Cg, which was very low in this study compared with the concentrations used in our study. Alternatively, we could not exclude the lack of expression of some molecular entity in HEK cells which might be responsible for Cg effect in macrophages. It is also important to point out that our TLR4 dependent effect of Cg is not due to contamination with LPS, which has been reported in Cg preparations [23]. Therefore, further studies will be necessary to understand whether Cg is a directly binding stimulator of TLR4, or alternatively whether Cg might be stimulating the release of an agonist of these receptors.

TLR4 agonists can induce activation of macrophages through MyD88-, TRIF-dependent pathways or both to produced pro-inflammatory cytokines [61–63]. Interestingly, our data suggest that Cg biased the production/release of TNF in a TLR4/CD14/MyD88 signaling pathway, while pro-IL-1β production depends on TLR4/CD14/TRIF pathway. Although TLR4/CD14/TRIF pathway is canonical signaling for Interferon-type I production, it might be considered an atypical signaling for pro-IL-1β production [64]. In this context, there is evidence that the activation of TRIF pathway seems to be downstream of TLR4 internalization into the endosomal network [65]. However, the production of TRIF dependent IL-1β by Cg-stimulated macrophages was not reduced by an actin polymerization inhibitor (Additional file 7), ruling out that Cg-induced endocytosis of the TLR4 is essential for this signaling functions. Therefore, further studies will be necessary to completely understand the molecular basis of Cg-stimulation of TLR4/TRIF pathway.

The signaling pathway mediated by TLR4/TRIF to the production of IL-1β in Cg-stimulated macrophage was demonstrated to activate tyrosine kinase Syk that in turn promotes ROS production. This signaling was previously undescribed for Cg-induced pro-IL-1β production by macrophages, although the involvement of Syk in Il1b expression has been described by different stimuli, such as dengue virus, beta-glucans, serum amyloid A and others [66–68]. Importantly, neither Syk nor ROS signaling plays a role for Cg-induced TNF production by macrophages, reinforcing the existence of two distinguish/independent pathways triggered by Cg/TLR4 that drives TNF or IL-1β, respectively. Although we did not investigate the final mechanisms by which Cg-triggered ROS induces pro-IL-1β expression, there is recent evidence showing that ROS drive the expression of pro-IL-1β through an increase in the transcriptional factor HIF-1α [28].

It is well accepted that IL-1β production depends on two distinct cellular signals [69, 70]. The first signal, which is normally driven by the activation of pattern recognition receptors (e.g. LPS through TLR4), culminates in the production of *Il1b* mRNA/pro-IL-1β [71]. The second signal is responsible for inflammasome activation that in turn convert pro-IL-1β into mature IL-1β [72, 73]. We showed here that Cg alone serves as these two signals since it was able to induce peritoneal macrophages to produce both pro-IL-1β and NLRP3-dependent IL-1β. Noteworthy, LPS is not able to induce mature IL-1β release by peritoneal macrophages, as Cg. This might indicate that there are additional components of Cg-induced NLRP3 activation and mature IL-1β production besides the stimulation of TLR4. Despite extensive studies on NLRP3 inflammasome activation by several cellular stimuli, [74, 75] its activation by Cg was not investigated yet. Here, we proposed that Cg activates this inflammasome via the convergent/canonical signal, the K+ efflux [42]. As the main K+ efflux upstream mechanisms for Cg-induced NLRP3 activation, it was found an important role for Panx1 and P2X7 receptors. We hypothesized that Cg initially promoted the opening of pannexin-1 channel for extrusion of ATP. Subsequently, ATP through P2X7 might drive K+ efflux. This hypothesis is in accordance with the activation of NLRP3 in human monocytes by TLR2 agonists and mouse macrophages by nanoparticles [44]. At this point, an important question that emerges from our study is how Cg drives Panx1 activation? In this context, there is evidence that intracellular LPS stimulates NLRP3 activation through the cleavage of Panx1 [76]. Furthermore, Panx1 cleavage opens its pore promoting the release of small amounts of ATP, that in turn activates P2X7 [76]. Although the levels of eATP in peritoneal macrophages after Cg stimulation are relatively low compared with the levels used for the activation of P2X7 by exogenous added ATP, we can speculate that Cg might decrease the threshold of ATP necessary to drive P2X7 opening as observed with intracellular LPS [76].

The identification of the pivotal role of NLRP3 inflammasome for Cg-induced macrophages production of IL-1b lead us to investigate whether this molecular mechanism would be important for Cg-induced in vivo inflammation. It has been demonstrated that Cg supplied in the drink water promotes spontaneous ulcerative lesions in the large intestine of rabbits, mice, rats and guinea pigs which resemble human ulcerative colitis [77, 78]. Supporting, it has been shown that in human intestinal epithelial cells, carrageenan triggers an inflammatory cascade, which seems dependent on TLR4 pathway [79, 80]. Furthermore, a randomized human trial showed that Cg enhances relapse in remission-patients with ulcerative colitis [81]. Our findings pointed out a crucial role for the canonical NLRP3 inflammasome in Cg-induced colitis in mice. Although the contribution of NLRP3 for Inflammatory Bowel Disease is controversial, [82] there is evidence suggesting its pro-inflammatory role in different experimental models of colitis [83, 84]. Nevertheless, the further understanding of the molecular signaling pathways stimulated by Cg might be useful to the identification of new targets for treating human intestinal diseases.

## Conclusion

In summary, the present study provides novel evidence for the molecular basis of Cg-induced cytokines production by macrophages. For instance, Cg requires TLR4/CD14/MyD88 signaling to stimulate TNF production, whereas TLR4/CD14/TRIF/SYK/ROS promotes pro-IL-1β. Furthermore, we unravel a critical role of the canonical NLRP3 inflammasome in Cg-induced IL-1β secretion and colitis, which is an important discovery on the pro-inflammatory properties of this sulfated polysaccharide for pre-clinical studies.

## Supplementary information

**Additional file 1.** Cg, but not LPS, induced IL-1β production by naive peritoneal macrophages. **(A, B)** Peritoneal macrophages were stimulated with Cg (300 μg/ml), Lipopolysaccharides (LPS - 1 μg/ml – 4 h) or medium. After treatments the supernatant were collect to quantify the IL-1β and TNF production by Elisa. Data are represent the mean ± SD of four independent experiments compared Control vs Treatments groups to determine the level of statistical significance (*p < 0.05; **p < 0.01; ***p < 0.001; ns, not significant).

**Additional file 2.** Neither reactive oxygen species nor Syk are involved in TNF production/release by Cg-stimulated macrophages. (A, B) Peritoneal macrophages were pre-incubated with N-acetylcysteine (3 NAC - 3 mM) or with selective inhibitor of Syk (iSyk, 1, 3 μM - 30 min) and then stimulated with Cg (300 μg/ml). After 6 h, the supernatants were collected for TNF quantification by Elisa. Data are represent the mean ± SD of four independent experiments compared WT (Cg) vs Treatments groups to determine the level of statistical significance (ns, not significant).

**Additional file 3.** Cg-induced TNF released by macrophages did not involved Casp1/11 neither Inflammasome platform. **(A)** Peritoneal macrophages were pre-incubated with selective inhibitor of Casp1 (Z-YVAD; 25, 50 μM - 30 min) and then stimulated with Cg (300 μg/ml). After indicated times, the supernatants were collected for TNF quantification by Elisa. **(B, C)** Peritoneal macrophages harvested from naive WT, *Casp1* 1^-*/−*^, *Casp1/11*^*−/−*^, *Casp11Tg*, *Nlrp3*^*−/−*^*, Nlrc4*^*−/−*^*, Pycard*^*−/−*^ mice were stimulated with Cg (300 μg/ml) or medium. After indicated time points, the supernatants were collected for quantification of TNF by Elisa. Data are represent the mean ± SD of four independent experiments compared WT vs Knockout/Treatments groups to determine the level of statistical significance (ns, not significant).

**Additional file 4.** Potassium efflux is not involved in Cg-induced TNF production by macrophages. **(A)** Peritoneal macrophages were maintained in regular medium or high-concentrated KCl (25 mM) medium and then stimulated by Cg (300 μg/ml). At indicated time points, the supernatants were collected for quantification of TNF by Elisa. Data are represent the mean ± SD of four independent experiments compared WT (Cg) vs Treatments groups to determine the level of statistical significance (ns, not significant).

**Additional file 5.** Osmolarity of Cg solutions. **(A)** The osmolarity of the medium was measured in the presence of different concentrations of Cg using an osmometer.

**Additional file 6.** The activation of P2X7/Panx1 channels is not involved in Cg-induced TNF production by macrophages. **(A)** Peritoneal macrophages were pre-incubated with a selective inhibitor of P2x7 (iP2x7) (10 uM - 30 min) and then stimulated with Cg (300 μg/ml). After 6 h, the supernatants were collected for TNF quantification by Elisa. **(B)** Peritoneal macrophages harvested from naive WT or *P2rx7*^*−/−*^ mice were stimulated with Cg (300 μg/ml) or medium. At indicated time points, the supernatants were collected for quantification of TNF by Elisa. **(C)** Peritoneal macrophages were pre-incubated with carbenoxolone (Cbx, 50 uM - 30 min) and then stimulated with Cg (300 μg/ml). At indicated times, the supernatants were collected for TNF quantification by Elisa. **(D)** Peritoneal macrophages harvested from naive WT or *Panx1*^*−/−*^ mice were stimulated with Cg (300 μg/ml) or medium. At indicated time points, the supernatants were collected for quantification of TNF by Elisa. Data are represent the mean ± SD of four independent experiments compared WT vs Knockout/Treatments groups to determine the level of statistical significance (ns, not significant).

**Additional file 7. **Role of phagocytosis on Cg-induced cytokines production by peritoneal macrophages **(A, B)** Peritoneal macrophages were pretreated with Cytochalasin D (30 μg/ml) and then stimulated by Cg (300 μg/ml). The supernatants were collected after 12 h of Cg stimulation for quantification by Elisa. Data are represent the mean ± SD of four independent experiments compared WT (Cg) vs Treatments groups to determine the level of statistical significance (*p <  0.05; **p <  0.01; ***p < 0.001; ns, not significant).

## Data Availability

All data generated or analysed during this study are included in the article.

## Abbreviations

ATP: Adenosine triphosphate
ASC: Apoptosis-associated Speck-like protein containing a Caspase-recruitment domain
β: Beta
Cg: Carrageenan
μg: Microgram
μL: Microliter
IL-1: Interleukin 1beta
LPS: Lipopolissacarídeo de *Escherichia coli*
NLRC4: IL-1β converting enzyme Protease-Activating Factor
NLRP3: NOD-like receptor family, pyrin domain containing 3
P2XR7: P2X purinoceptor 7
ROS: Reactive oxygen species
Syk: Spleen tyrosine kinase
TNF: Tumor necrosis factor
