# Supplementary figures and images for: Molecular basis of carrageenan-induced cytokines production in macrophages
Source: Cell Commun Signal. 2020 Sep 7;18:141. doi: 10.1186/s12964-020-00621-x (PMC7487827; doi:10.1186/s12964-020-00621-x)

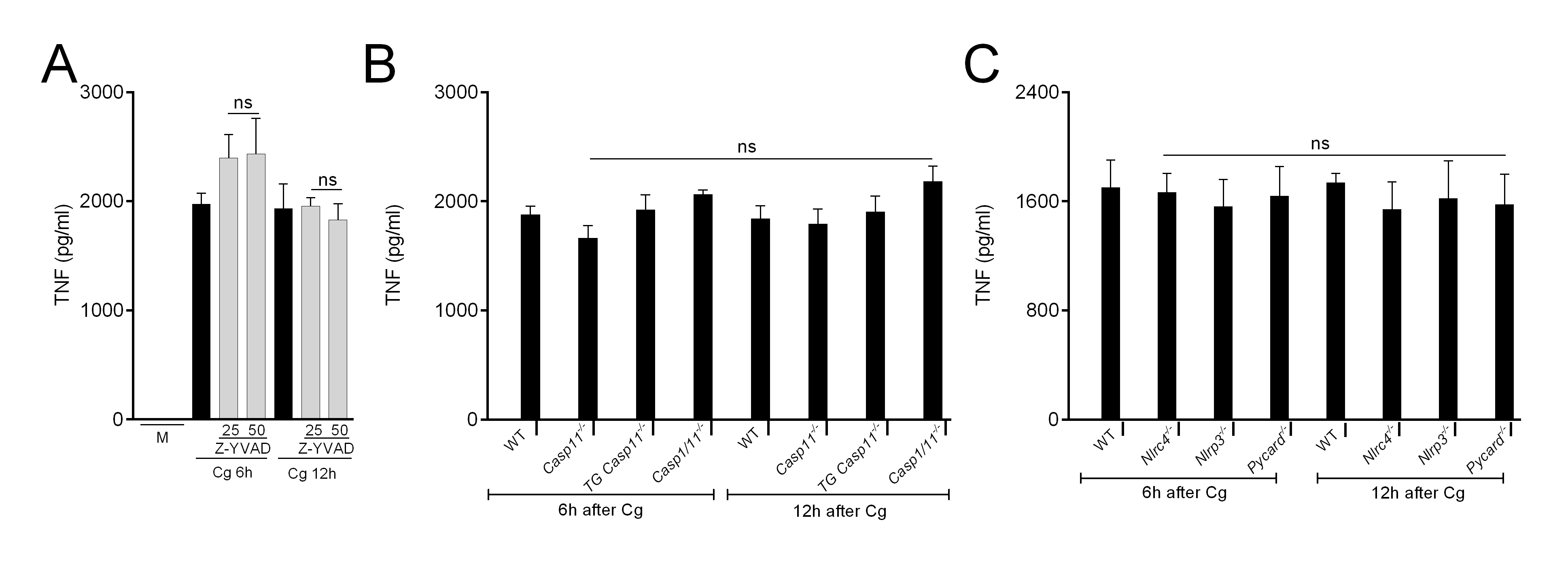

Supplement: Supplementary file 4 — Additional file 3. Cg-induced TNF released by macrophages did not involved Casp1/11 neither Inflammasome platform. (A) Peritoneal macrophages were pre-incubated with selective inhibitor of Casp1 (Z-YVAD; 25, 50 μM - 30 min) and then stimulated with Cg (300 μg/ml). After indicated times, the supernatants were collected for TNF quantification by Elisa. (B, C) Peritoneal macrophages harvested from naive WT, Casp1 1-/−, Casp1/11−/−, Casp11Tg, Nlrp3−/−, Nlrc4−/−, Pycard−/− mice were stimulated with Cg (300 μg/ml) or medium. After indicated time points, the supernatants were collected for quantification of TNF by Elisa. Data are represent the mean ± SD of four independent experiments compared WT vs Knockout/Treatments groups to determine the level of statistical significance (ns, not significant). [file 12964_2020_621_MOESM4_ESM.tif]
